# Variable retention harvesting influences belowground plant-fungal interactions of *Nothofagus pumilio* seedlings in forests of southern Patagonia

**DOI:** 10.7717/peerj.5008

**Published:** 2018-07-06

**Authors:** Rebecca E. Hewitt, Donald Lee Taylor, Teresa N. Hollingsworth, Christopher B. Anderson, Guillermo Martínez Pastur

**Affiliations:** 1 Center for Ecosystem Science and Society, Northern Arizona University, Flagstaff, AZ, United States of America; 2 Institute of Arctic Biology, University of Alaska—Fairbanks, Fairbanks, AK, United States of America; 3 Department of Biology, University of New Mexico, Albuquerque, NM, United States of America; 4 Pacific Northwest Research Station, Boreal Ecology Cooperative Research Unit, US Forest Service, Fairbanks, AK, United States of America; 5 Centro Austral de Investigaciones Científicas (CADIC), Consejo Nacional de Investigaciones Científicas y Técnicas (CONICET), Ushuaia, Tierra del Fuego, Argentina; 6 Instituto de Ciencias Polares, Ambiente y Recursos Naturales (ICPA), Universidad Nacional de Tierra del Fuego (UNTDF), Ushuaia, Tierra del Fuego, Argentina

**Keywords:** Lenga, Dispersed retention, Ectomycorrhizal fungi, Tierra del fuego, Forest sustainability, Recruitment, Silviculture, Aggregate retention

## Abstract

**Background:**

The post-harvest recovery and sustained productivity of *Nothofagus pumilio* forests in Tierra del Fuego may be affected by the abundance and composition of ectomycorrhizal fungi (EMF). Timber harvesting alters EMF community structure in many managed forests, but the impacts of harvesting can vary with the management strategy. The implementation of variable retention (VR) management can maintain, increase, or decrease the diversity of many species, but the effects of VR on EMF in the forests of southern Patagonia have not been studied, nor has the role of EMF in the regeneration process of these forests.

**Methods:**

We evaluated the effects of VR management on the EMF community associated with *N. pumilio* seedlings. We quantified the abundance, composition, and diversity of EMF across aggregate (AR) and dispersed (DR) retention sites within VR managed areas, and compared them to primary forest (PF) unmanaged stands. EMF assemblage and taxonomic identities were determined by ITS-rDNA sequencing of individual root tips sampled from 280 seedlings across three landscape replicates. To better understand seedling performance, we tested the relationships between EMF colonization, EMF taxonomic composition, seedling biomass, and VR treatment.

**Results:**

The majority of EMF taxa were Basidiomycota belonging to the families Cortinariaceae (*n* = 29), Inocybaceae (*n* = 16), and Thelephoraceae (*n* = 8), which was in agreement with other studies of EMF diversity in *Nothofagus* forests. EMF richness and colonization was reduced in DR compared to AR and PF. Furthermore, EMF community composition was similar between AR and PF, but differed from the composition in DR. EMF community composition was correlated with seedling biomass and soil moisture. The presence of *Peziza depressa* was associated with higher seedling biomass and greater soil moisture, while *Inocybe fibrillosibrunnea* and *Cortinarius amoenus* were associated with reduced seedling biomass and lower soil moisture. Seedling biomass was more strongly related to retention type than EMF colonization, richness, or composition.

**Discussion:**

Our results demonstrate reduced EMF attributes and altered composition in VR treatments relative to PF stands, with stronger impacts in DR compared to AR. This suggests that VR has the potential to improve the conservation status of managed stands by supporting native EMF in AR. Our results also demonstrate the complex linkages between retention treatments, fungal community composition, and tree growth at individual and stand scales.

## Introduction

Southern Patagonia is globally recognized for the conservation value of its extensive forests (Olson & Dinerstein, 2002; Mittermeier et al., 2003). These forests also constitute regionally important economic and cultural resources as working timberlands and rangelands (Peri et al., 2016a; Martínez Pastur et al., 2017). During early European colonization, Patagonian forests were subjected to extensive clearing, burning, and grazing, but in the last several decades new silviculture techniques have been implemented (Gea-Izquierdo et al., 2004) that attempt to achieve greater forest sustainability (Gamondès Moyano, Morgan & Martínez Pastur, 2016). Variable retention (VR) management has been adapted from timber practices in the Northern Hemisphere (Franklin et al., 1997) and implemented in southern Patagonia as a way to reconcile the goals of preserving forest biodiversity and production values (Soler et al., 2015; Soler et al., 2016). The VR management strategy in our study system in Tierra del Fuego leaves 30% of a given managed area as intact patches of primary or secondary forest called “aggregate retention” (AR). These patches are located within a harvested area called “dispersed retention” (DR), where 20% of the basal area remains standing to serve as seed trees, regeneration shelter, or for conservation purposes.

*Nothofagus pumilio* (Popp. et Endl.) Krasser (commonly named lenga) is one of the dominant canopy tree species and the most important timber species in the deciduous temperate forests of the Magellanic subpolar ecoregion (Martínez Pastur et al., 2000). Tree seed banks do not persist in the soils of *N. pumilio* forests (Cuevas & Arroyo, 1999) and, thus, seedbed and environmental conditions at the time of dispersal and germination are key determinants of forest regeneration after timber harvesting (Martínez Pastur et al., 2011). Within a VR management system, seed dispersal and germination do not limit forest regeneration, due to close proximity to seed sources (less than twofold the dominant tree height) and adequate seedbed conditions, regardless of the degree of canopy cover remaining after harvest (Martínez Pastur et al., 2014; Torres et al., 2015). Instead, lenga establishment and performance is influenced by environmental modifications resulting from forest harvesting, such as changes in soil moisture and light levels (Martínez Pastur et al., 2007; Martínez Pastur et al., 2011), and potentially biotic factors, such as availability of compatible symbiotic fungi.

Like other *Nothofagus* species, lenga relies upon an obligate symbiosis with ectomycorrhizal fungi (EMF) (Smith & Read, 2008). In general, EMF colonize the fine root systems of host plants, improving the plant’s ability to access soil resources including water and nutrients and in turn receive carbon in the form of photosynthate (Smith & Read, 2008). This symbiosis is considered a mutualism; yet, at the seedling phase it can have either beneficial or detrimental effects on seedling performance, depending on the taxon-specific carbon costs and nutrient benefits conferred to the seedling by the mycobiont (Jones & Smith, 2004; Koide et al., 2008; Van der Heijden & Horton, 2009; Hoeksema et al., 2010). Few ecological studies of fungal diversity have been carried out in Patagonia (but see Truong et al., 2017) and even less is known about root-associated EMF dynamics in Patagonian forests (but see Palfner, Canseco & Casanova-Katny, 2008; Nouhra et al., 2013); however, other understudied Southern Hemisphere forests have EMF host trees including *Nothofagus* (Orlovich & Cairney, 2004; Tedersoo et al., 2008; Dickie, Richardson & Wiser, 2009; Tedersoo et al., 2009; Horton et al., 2013), and collectively these studies inform our interpretation of diversity patterns and plant-fungal interactions in Tierra del Fuego.

In managed forests around the world, successful seedling establishment has been linked to adequate EMF inoculum, fungal species assemblage, and diversity (Amaranthus & Perry, 1987; Perry, Molina & Amaranthus, 1987; Perry et al., 1989). Timber harvesting can alter EMF community structure, decrease EMF inoculum potential, and reduce fine root colonization levels (Parke, Linderman & Trappe, 1984; Borchers & Perry, 1990; Hagerman et al., 1999; Jones, Durall & Cairney, 2003). These findings are particularly evident after clear-cutting, where host plants have been killed and the survival of EMF species on mature trees is minimal and therefore limited in the timeframe of natural or out-planted seedling recruitment (Dahlberg et al., 2001; Simard, 2009). However, with VR management, AR patches may act as islands of ecological integrity where diversity, structure, and function are preserved. In northern forested ecosystems both AR (Kranabetter, De Montigny & Ross, 2013) and individual seed trees can maintain EMF colonization and diversity after harvest (Outerbridge & Trofymow, 2004; Cline, Ammirati & Edmonds, 2005) with positive effects on seedling performance (Cline, Vinyard & Edmonds, 2007). Yet, other studies have shown that seed trees alone fail to maintain EMF diversity for upwards of several decades after harvesting (Varenius, Lindahl & Dahlberg, 2017). In comparison to EMF studies from the Northern Hemisphere, relatively little is known about post-harvest EMF dynamics in Southern Hemisphere forests. In New Zealand ten years after harvest, Dickie, Richardson & Wiser (2009) reported reduced occurrence of EMF roots and altered EMF composition in *Nothofagus* forests. Together with post-harvest observations from the Northern Hemisphere, these findings suggest that there may be strong effects of harvesting disturbance on EMF-seedling dynamics. In this context, EMF-seedling dynamics in managed Patagonian forested ecosystems could be crucial for understanding the post-harvest recovery and sustainability of forestry practices.

In this study, we sought to: (i) understand how belowground mycobiont diversity and composition was impacted by VR management treatments (AR and DR) in comparison to primary forest (PF) stands, (ii) determine whether variation in lenga biomass was correlated with EMF factors (% colonization, structure, and occurrence of specific taxa) and environmental factors (soil moisture) that are known to be influenced by harvesting, (iii) evaluate whether our results support the concept that AR patches act as refugia for EMF, thus contributing to biodiversity conservation in southern Patagonian forests, and (iv) compare the taxonomic diversity observed in our study to EMF reported in other Southern Hemisphere forests. We tested four hypotheses: (i) seedling root colonization will decline from PF to AR to DR, (ii) fungal communities will be more similar between PF and AR than PF and DR, (iii) fungal composition will be related to seedling biomass with larger seedlings associated with taxa in PF stands, and (iv) dominant EMF taxa observed in association with *N. Pumillo* seedlings will be similar to those found in other Southern Hemisphere forests. This study builds on an extensive body of research investigating post-harvest dynamics in Northern Hemisphere forests and extends this research to the Southern Hemisphere by providing a first investigation of these dynamics in a VR-managed stands in Patagonia. As such, these findings are expected to contribute to our understanding of EMF diversity patterns in an underexplored ecosystem and provide further evidence of the impacts of VR harvesting on biodiversity in Tierra del Fuego’s *Nothofagus* forests.

## Materials & Methods

### Study site

This study was carried out in the Argentine portion of Tierra del Fuego Island, where working timberlands constitute c.a. 220,000 ha of monospecific lenga stands (Collado, 2001). We studied three old-growth *N. pumilio* stands and adjacent areas of VR timber management (with AR and DR treatments) at Los Cerros Ranch (54°18′S, 67°49′W). Primary forest stands are 5–10 ha with uneven age structure and older trees aged up to 400 years old. The PF stands presented a closed canopy with a sparse understory of relatively few woody and herbaceous plants (Lencinas et al., 2008). Managed stands with the VR system include areas of AR, intact forest “islands” (60 m radius, one per hectare), surrounded by DR. In DR areas evenly dispersed seed trees maintain a basal area of dominant trees at 10–15 m^2^ ha^−1^ (Martínez Pastur et al., 2009). These sites are part of the *Parcelas de Ecología y Biodiversidad de Ambientes Naturales en Patagonia Austral* (*PEBANPA*), a long-term monitoring network in southern Patagonia (Peri et al., 2016b), and have been monitored for seedling recruitment dynamics since 2004.

### Seedling sampling and regeneration measurements

To characterize seedling EMF communities across timber management treatments in relation to seedling biomass, we sampled seedlings in three replicate sites of each PF, AR, and DR treatment. Logging occurred in the growing season of 2004–2005, and in 2012 we focused our sampling on young seedlings, most of which were under seven years old and established after logging. At each site we sampled 10 lenga seedlings along three randomly positioned 50 m transects in close proximity (10–50 m) to permanent PEBANPA network regeneration plots, collecting one seedling approximately every five meters, for a total of 30 seedlings per site and 90 seedlings per treatment. In all sites our sampling did not account for distance to the rooting zone of lenga trees and therefore represents the full variation of EMF-seedling dynamics. At one of PF sites we sampled an additional transect. In total, we sampled 280 seedlings across 28 transects and nine sites.

Harvested seedlings were transported back to the Agroforestry Lab at the *Centro Austral de Investigaciones Científicas* (CADIC-CONICET) in Ushuaia, Argentina. Shoots were separated from roots. Aboveground biomass was dried at 70 °C for 48 h and weighed. The age of the seedling was verified by counting leaf whirls (Martínez Pastur et al., 2011). Thirteen of 280 seedlings established before 2005, e.g., prior to timber harvest, across all three treatments (three in AR, seven in DR, and three in PF). We chose not to exclude these seedlings because they were relatively well distributed across treatments and comparative bi- and multi-variate analyses indicated that they did not impact statistical results.

We obtained lenga regeneration metrics including sapling (>1 year-old plants) and seedling (<1 year old plants) density in the permanent plots of the PEBANPA network at our sites (Peri et al., 2016b). Survey methods for lenga regeneration are fully described in Martínez Pastur et al. (2011). In brief, seedling and sapling density was surveyed in 18 regeneration plots of 1 m^2^ (500 × 20 cm) in each treatment (PF, AR, DR). We also measured volumetric soil water content (VSW) in the upper 10 cm of soil using a MP406 moisture probe (ICT, Armidale, Australia) at the beginning and end of the summer.

### EMF root colonization

The root system of each seedling was rinsed gently to remove soil, and then was cut into 5 cm segments. Root segments were dispersed in water in a gridded Petri dish under a dissecting microscope at 10–40× magnification. We used the gridline intercept method (Brundrett et al., 1996) to count the proportion of fine roots colonized by EMF in relation to the total number of root-gridline intersections. We randomly selected five healthy EMF-colonized root tips per seedling and preserved them in RNAlater (Ambion, Inc., Austin, Texas, USA).

### Molecular analysis of EMF composition

EMF root tips were rinsed with distilled water to remove excess RNAlater and then were lyophilized. Root tips were ground in lysis buffer with a motorized sterile pestle (Kontes, Rockwood, TN, USA). DNA was extracted from each root tip using the DNEasy Plant Mini Kit (*QIAGEN* Inc., Valencia, CA, USA) according to the manufacturer’s instructions with minor modifications suggested in Bent & Taylor (2010). Genomic DNA was amplified in 25 µl PCR reactions containing 25 mM MgCl2, 10 mM dNTPs, 50 µM forward primer ITS1-F (CTTGGTCATTTAGAGGAAGTAA (Gardes & Bruns, 1993)), 50 µM reverse primer ITS4 (TCCTCCGCTTATTGATATGC (White et al., 1990)), 10 mg/ml bovine serum albumin, Promega Go-Taq (Sigma-Aldrich, St. Louis, MO, USA), 5X Promega Green Taq buffer, and 5 µl of genomic DNA. Reaction mixes were prepared in 0.2 ml tubes and thermocycled in an MJ Research PTC-225 thermal cycler as follows: 96 °C for 3 min, 35 cycles of 94 °C for 30 s, 52 °C for 30 s, then 72 °C for 3 min, followed by 72 °C for 10 min. PCR products were gel checked and Sanger sequenced by Functional Biosciences (Madison, WI).

### Bioinformatics

Sanger sequences were trimmed, quality filtered, and grouped into operational taxonomic units (OTUs), synonymous with EMF taxa, as follows. Initial end-trimming (error rate below 0.1 in a 25 base window for both ends) and assembly of paired end reads (where available) were carried out in Codoncode Aligner 4.03. Sequences with fewer than 200 high quality base calls (phred > 20) were deleted and all remaining sequences were exported in fastq format. Next, a more stringent end-trimming was carried out using FASTQ Quality Trim program in Galaxy (Goecks, Nekrutenko & Taylor, 2010), with window sizes of 5, 20 and 40 and a phred threshold of 20. Sequences were converted to fasta format in Galaxy then aligned using Muscle (Edgar, 2004) in AliView (Larsson, 2014) to identify and manually remove primer sequences. We then used QIIME 1.9 (Caporaso et al., 2010) to cluster sequences into OTUs via open-reference with the USEARCH method (Edgar, 2010), a 95% sequence identity threshold, and the UNITE sh_refs_qiime_ver7_97_s_20.11.2016 database (Kõljalg et al., 2013). Singletons were kept at this step (min_otu_size 1). Representative sequences for each OTU were submitted to the RDP Naïve Bayesian classifier using the Warcup 2 ITS training set to estimate OTU taxonomies (Deshpande et al., 2016). We also searched GenBank using discontiguous mega-BLAST, with uncultured sequences excluded, to confirm, and in some cases improve identifications produced by RDP. OTU trophic guilds were inferred based on investigator knowledge and literature searches. All sequences are deposited in GenBank under accession numbers MH019772 –MH019959.

Following the taxonomic identification of EMF, OTUs were eliminated if they were saprotrophs, yeasts, wood decay fungi, pathogens, or if identification was uncertain. For further ordination analysis singletons (OTUs occurring once) were also eliminated to reduce the disproportionate influence of extremely rare taxa (McCune & Grace, 2001), resulting in a matrix of 49 OTUs × 228 seedlings.

### Data analysis

We conducted comparisons for stand metrics including basal area, soil moisture, and the regeneration metrics including seedling and sapling density for each VR treatment (Table 1). These comparisons were tested using one-way ANOVA and further pairwise comparisons using the Tukey test of honest significant difference (*p* < 0.05). All statistical analyses were performed in R (R Core Team, 2016) with the exception of EMF ordination analysis and rarefaction curves described below. The seedling biomass and colonization dataset and ectomycorrhizal community composition dataset are archived with Bonanza Creek LTER: doi: 10.6073/pasta/350b701beb4ac2ebd2ef3f29893e6ed5, http://www.lter.uaf.edu/data/data-detail/id/687; doi: 10.6073/pasta/df801ec9b7f3493b6eb3bf191124e41f, http://www.lter.uaf.edu/data/data-detail/id/686. Seedling recruitment and density data are available on PEBANPA databases through CADIC-CONICET Ushuaia.

**Table 1:** Site and EMF variables for primary forest and variable retention timber treatments (aggregate and dispersed retention). Data included show means followed by S.E.

	Primary forest	Aggregate retention	Dispersed retention
Mean basal area (m^2^/ha)^*^	94.89 ± 2.17^a^	71.94 ± 2.55^b^	8.5 ± 1.26^c^
Mean soil moisture (water/soil)^*^	21.30 ± 1.79^a^	19.42 ± 1.60^a^	34.48 ± 2.28^b^
Mean seedling density (thousand/ha)^*^	203 ± 107^a^	134 ± 83^a^	18 ± 8^b^
Mean sapling density (thousand/ha)^*^	157 ± 48^a^	251 ± 58^a^	86 ± 10^b^
Site EMF *α* diversity^*^	27.67 ± 1.22^a^	27.00 ± 1.53^a^	15.67 ± 2.85^b^
Seedling EMF *α* diversity^*^	1.73 ± 0.08^a^	1.43 ± 0.07^b^	1.15 ± 0.08^c^
Mean colonization (%)^**^	97.2 ± 0.01^ab^	99.0 ± 0.01^a^	95. 5 ± 0.01^b^

**Notes.**

* post hoc Tukey HSD at *p* < 0.05.

** post hoc Dunn Test at *p* < 0.05.

a, b, c indicate significant differences detected by post hoc tests.

### Analysis of EMF root colonization

To compare mean root colonization for each retention type we used the Kruskal–Wallis test with a post hoc Dunn Test of multiple comparisons with the Bonferroni correction with the R package dunn.test (Dinno, 2017). We used a non-parametric test because EMF colonization levels were strongly skewed due the large number of root systems colonized at high levels (mean colonization across treatments (97.2% ± 0.01 S.E.)), and regardless of data transformation the data did not meet the assumption of normality.

### Analysis of EMF community structure

For EMF taxa richness and evenness, we counted the EMF taxa occurring across all seedlings within each treatment to compute a total richness estimation for PF, AR, and DR areas. At the treatment level we also summed the number of species shared between each two-way and the full three-way comparison of treatments. To compute EMF α diversity at the seedling level, we counted the number of EMF taxa associated with each seedling from our sequence dataset. We tested for significant treatment differences in seedling-level α diversity using one-way ANOVA, and tested for differences between two-way comparisons using the Tukey test of honest significant difference (*p* < 0.05). We used the same model structure to test for treatment differences in α diversity at the site level. Furthermore, we computed sample-based abundance rarefaction curves for each treatment to assess variation in richness across treatments. Diversity statistics for rarefaction curves were calculated using EstimateS (Colwell, 2013), where we used 100 randomization runs and randomized the samples without replacement.

To evaluate patterns in seedling- and transect-level EMF composition (Kruskal & Wish, 1978; McCune & Mefford, 2011) we performed Nonmetric Multidimensional Scaling (NMDS) ordinations. We used the Sorensen (Bray–Curtis) distance measure to represent compositional dissimilarity. We identified the solution with the lowest stress relative to the number of axes (McCune & Grace, 2002). Finally, we calculated a post hoc proportion of variance represented by each axis by calculating the squared Pearson correlation (*r*^2^) between distances in the original distance metric and pair-wise distances of objects in the ordination space (McCune & Grace, 2002).

Preliminary analysis of composition associated with individual seedlings suggested a 1-dimensional solution, which would be highly unstable and not significantly different than random. We therefore excluded all OTUs that occurred on less than four seedlings. This resulted in a final matrix of 26 OTUs × 206 seedlings. Our final matrix was transformed using Beal’s smoothing to relieve the “zero truncation problem” (Beals, 1984), which is common with heterogeneous data with a large number of zeros. We exported three ordination axes and used those as representations of EMF composition in Random Forest and liner mixed-effects model analyses described below (Fig. S1).

We combined EMF composition on individual seedlings across each transect for ordination analysis of EMF composition in relation to environmental factors. Pooling EMF composition data at the transect level allowed us to evaluate variation in composition within and across sites. We correlated this main OTU matrix with a secondary environmental matrix and expressed high correlations (*r* ≥ 0.20) with a biplot overlay to indicate the direction and magnitude of significantly correlated environmental variables. The secondary matrix was made up of seven environmental variables: treatment (PF, AR, DR), site (*n* = 9), volumetric soil water content, average seedling biomass (g), average % root colonization, and average age of seedlings across the transect. We tested whether EMF composition varied by VR treatments using Multiple-Response Permutation Procedure (MRPP) (Berry, Kvamme & Mielke, 1983) and tested whether the abundance of any taxon was an indicator of the VR treatments with Indicator Species Analysis (Dufrene & Legendre, 1997). All ordination analyses of EMF communities were performed in PC-ORD 6.0 (MJM Software Design, Gleneden Beach, OR, USA).

### Analysis of relationships between seedling biomass and EMF metrics

To evaluate the importance of retention treatments, seedling age, and EMF variables (colonization, richness, and composition) in predicting seedling biomass, we used Random Forest (RF) regression trees in the randomForest package in R (Liaw & Wiener, 2015). RF is an ensemble decision tree method that uses an algorithmic approach to make predictions based on the input variables (Breiman, 2001b). The RF approach allowed us to include all our measured variables, including untransformed root colonization and biomass data because RF can accurately predict variable response with data that is not normally distributed. We used RF to calculate variable importance scores for treatment, seedling, and fungal variables when predicting seedling biomass. We built 500 regression trees using random samples of the 206 observations. At each node in the tree, two predictor variables were chosen at random from the seven explanatory variables: management treatment, seedling age, and EMF composition represented by three NMDS axes, EMF richness, and % root colonization. For each predictor, RF computes the prediction error (mean square error). The difference between the classification of observed bootstrap data and the classification of permuted data is averaged across all trees and divided by the standard error (Breiman, 2001a; Cutler et al., 2007).

We also created linear mixed-effects models using the nlme package (Pinheiro et al., 2018) to explore the fixed-effects of timber management treatment, seedling age, EMF composition represented by three NMDS axes, and EMF richness on seedling biomass with seedling nested in transect and site as a random factor. We evaluated data distributions and correlations between all predictor variables (habitat, age, root colonization, EMF richness, Axis1, Axis2, and Axis3) with a threshold spearman correlation of 0.4. None were excluded based on this criterion. Seedling biomass was log-transformed.

To further evaluate whether the presence of specific EMF taxa affected seedling biomass we correlated the transect-level species matrix with itself yielding a correlation matrix of OTUs that strongly correlated (>0.20) with each axis (Hollingsworth et al., 2013). We then used a simple ANOVA to evaluate differences in seedling biomass for seedlings that were colonized (yes/no) by each of these strongly correlated taxa.

## Results

### VR impacts on seedling recruitment

Forest structure significantly varied among treatments. Basal area was greatest in PF, followed by AR, and then DR sites (*F* = 248.14, *p* < 0.01, Table 1) due to the removal of trees during timber harvesting. Harvesting treatment greatly influenced soil moisture (*F* = 15.66, *p* < 0.01). Dispersed retention sites had greater soil moisture compared to PF and AR sites (Table 1). We found marginal differences in seedling density by treatment (*F* = 2.73, *p* = 0.08, Table 1). Primary forest and AR sites had higher seedling densities compared to DR sites. Seedling densities ranged from 0 to 166 thousand ha^−1^ in DR sites, 0 to 786 thousand ha^−1^ in AR, and 0 to 980 thousand ha^−1^ in PF sites. Sapling density followed the same trend, but with significant differences among treatments (*F* = 6.27, *p* < 0.01, Table 1). Sapling densities ranged from 35 to 203 thousand ha^−1^ in DR sites, 78–620 thousand ha^−1^ in AR, and 6–477 thousand ha^−1^ in PF sites.

### VR impacts on EMF root colonization

On average, colonization rates were high (97.2% ± 0.01 S.E.) across all treatments. EMF colonization varied from 38% to 100% among seedlings. Colonization levels varied with timber management treatment (Kruskal–Wallis *χ*^2^ = 11.63, *p* < 0.01). On average AR sites had the highest mean colonization, which was significantly higher than colonization levels in the DR sites, but similar to those in PF sites (Table 1).

### EMF taxonomic diversity

Our sequencing effort yielded 188 OTUs, 89 of which were EMF or likely EMF (Tables S1 and S2). The most abundant OTUs in the sequencing dataset were taxa in the EMF and likely EMF trophic niche with 17 EMF taxa occurring in the Phylum Ascomycota (Subphylum Pezizomycotina) and 72 in the Basidiomycota (Subphylum Agaricomycotina). All of the fungi in the Basidiomycota were in class Agaricomycetes and belonged to the orders Agaricales, Boletales, Cantharellales, Russulales, Sebacinales, and Thelephorales. The families Cortinariaceae (*n* = 29), Inocybaceae (*n* = 16), and Thelephoraceae (*n* = 8) were the most species rich. Despite the Basidiomycota showing higher EMF taxonomic diversity, the most abundant OTUs were ascomycetes (Table S1).

### VR impacts on EMF taxa richness and occurrence

Summaries of our sequence data indicate that of the EMF taxa, 52 occurred in PF, compared to 53 in AR, and only 30 in DR. Twenty-eight taxa observed in PF stands also occurred in AR, while only 13 PF taxa occurred in DR. Seedlings from AR and DR areas shared 15 taxa. Overall, 10 of 89 taxa occurred in all treatments (Table S2). Despite some of the dominant taxa occurring across all VR timber treatments, the abundances of many of these dominants varied with VR treatment. For example, the most abundant taxon, OTU 1 *Peziza depressa,* occurred primarily in the DR areas. Conversely, some dominant *Cortinarius, Tomentella*, and *Inocybe* species had low abundances or were not observed in the DR sites (Table S2).

**Figure 1:**
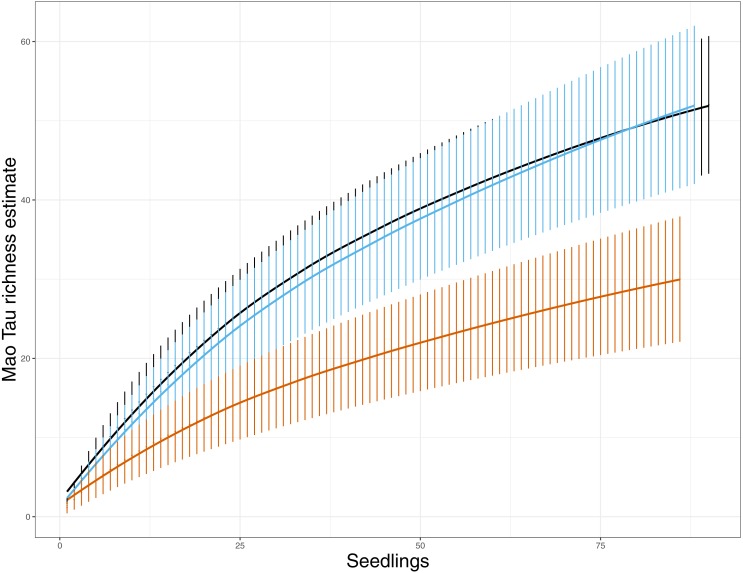
EMF species richness for both variable retention treatments and primary forest. Values are Mao Tau abundance based richness estimates with 95% CI (Colwell, Mao & Chang, 2004). Primary forest, black; aggregate retention, blue; dispersed retention, orange. The rarefaction curves indicate that richness was reduced in dispersed retention compared to aggregate retention and primary forest stands.

At the seedling level, EMF richness was low, ranging from one taxon to three taxa per seedling (Table 1). These richness estimates include EMF that were observed once (e.g., singletons). Seedlings in PF stands had significantly greater EMF richness than seedlings in AR and DR, and seedlings in AR showed greater richness than seedlings in DR areas (*F* = 15.00, *p* < 0.001, Table 1). These patterns of α diversity shifted slightly when we assessed the richness at the site level. Here again richness varied by treatment (*F* = 11.16, *p* = 0.01) with PF and AR sites showing greater richness than DR sites (Table 1), but no significant difference in richness was detected between AR and PF. Richness ranged from 25 to 29 taxa detected in PF sites, 24–29 taxa in AR sites, and 10–19 in DR sites. This was supported by the species rarefaction curves, which indicated that EMF species richness was lower in DR sites (Fig. 1). The shape of the curves also suggest that we did not saturate EMF diversity with our sampling in any of the treatment categories, but we likely came closer in the DR sites.

### VR impacts on EMF composition

**Figure 2:**
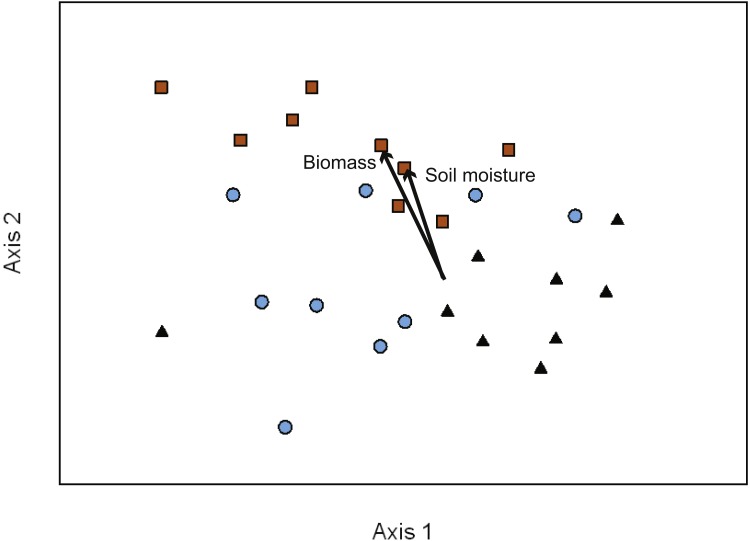
NMDS ordination of EMF communities associated with each transect (10 seedlings per transect). Symbols represent the two treatments in the variable retention timber management system and primary forests stands. Primary forest, black triangles; aggregate retention, blue circles; dispersed retention, orange squares. Proportion of variance explained by each axis: Axis 1 = 21.3%, Axis 2 = 18.7%, and Axis 3 = 35.2%. The vectors indicate the magnitude and direction of the relationship. The ordination indicates that differences in composition were greatest between dispersed retention and aggregate retention or primary forest stands.

To determine the effects on EMF composition of VR management treatments (AR and DR) in comparison to PF stands we used NMDS ordination to visualize the variation in taxon abundance and occurrence within and across our study sites. Three major gradients captured ∼75% of the variance in EMF composition across transects (Axis 1 = 21.5%, Axis 2 = 18.4%, and Axis 3 = 35.3% of the variation). The NMDS ordination had a final stress of 14.45 with a final instability of 0.00046 after 278 iterations (Fig. 2). Soil moisture and seedling biomass were correlated with fungal composition along Axis 2 (seedling biomass *r*^2^ = 0.47; soil moisture *r*^2^ = 0.45). Additionally, there was a relatively strong visual pattern of differentiation in composition between treatments (Fig. 2). This was confirmed by MRPP analysis (*T* =  − 7.43, *A* = 0.08, *p* < 0.01) with the strongest differentiation between DR and AR (*T* =  − 6.42, *A* = 0.08, *p* < 0.01) or PF (*T* =  − 6.72, *A* = 0.10, *p* < 0.01). There was no significant difference in species composition between PF and AR sites (*T* =  − 1.57, *A* = 0.02, *p* = 0.07). Indicator species analysis showed that the abundance of OTU14, *Timgrovea ferruginea*, was an indicator of DR (IV = 42.70, *p* = 0.03), and OTU34, *Cortinarius cycneus*, was an indicator of AR (IV = 58.30, *p* < 0.01).

### Relationships between seedling biomass and EMF metrics

**Figure 3:**
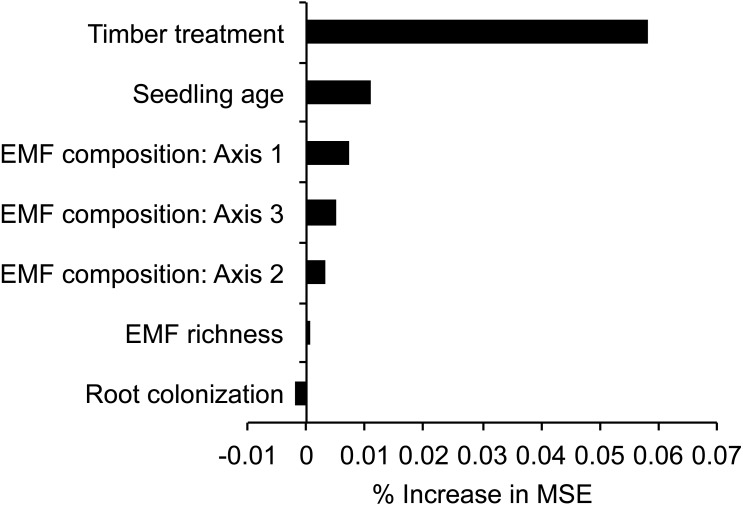
Variable importance scores based on Random Forest regression trees explaining seedling biomass in relation to variable retention timber management treatment, seedling and fungal variables. Scores are derived from the increase in the mean square error (% increase MSE) when a variable is permuted. Axis1, 2, and 3 were derived from NMDS ordination of EMF composition of each seedling. Variable retention treatment and seedling age were more important predictors of seedling biomass than EMF variables.

Random Forest analysis of individual seedlings indicated that treatment (PF, AR, or DR) was the most important variable predicting seedling biomass, followed by seedling age, EMF composition, EMF richness, and % root colonization (Fig. 3). Supporting these results, the mixed-effects model analysis also indicated that seedling biomass was affected by treatment (*χ*^2^ = 144.73, *p* < 0.01) and seedling age (*χ*^2^ = 33.07, *p* < 0.01), while EMF composition and richness were not significantly important to explaining variation in seedling biomass (Table 2). The mixed-effects model accounted for 70.7% of the variance explaining log-transformed seedling biomass (${R}_{\mathrm{marginal}}^{2}=0.71$, ${R}_{\mathrm{conditional}}^{2}=0.76$). Mixed-effects model parameter estimates indicate that seedlings from the AR and DR sites had greater biomass than seedlings harvested from the PF sites (PF 0.06 g ± 0.00 S.E., AR 0.11 g ± 0.01 S.E., DR 0.56 g ± 0.04 S.E., Table 2). The mean age of seedlings in PF was 4.30 years ± 0.12 S.E., slightly younger than in DR 4.58 years ± 0.16 S.E. and significantly older than in AR (3.76 years ± 0.16 S.E.) (*F* = 8.07, *p* < 0.01, where D-A diff 0.82 *p* < 0.01, PF-A diff 0.54 *p* = 0.02, PF-D diff −0.28 *p* = 0.36). Although seedling biomass was higher in the DR, seedling and sapling density were lower in DR sites, than AR or PF (Table 1).

**Table 2:** Linear mixed-effects model coefficients and associated S.E. explaining log- transformed seedling biomass. The intercept or reference level is set as the log-transformed seedling biomass for seedlings occurring in primary forest. Bolded *p*-values indicate parameters that were significanlty related to seedling biomass.

Variable	Value	S.E.	*p*-value
Primary forest	−1.55	0.08	**<0.01**
Aggregate retention	0.25	0.07	**0.01**
Dispersed retention	0.81	0.07	**<0.01**
Seedling age	0.07	0.01	**<0.01**
Axis1	0.04	0.03	0.21
Axis3	0.05	0.04	0.19
Axis2	0.01	0.03	0.82
Seedling EMF *α* diversity	0.02	0.03	0.40

### Taxon-specific relationships between EMF and seedling biomass

Five OTUs were correlated with Axis 2 (Axis 2 = 18.7% of 75% total variance explained), the same axis that was correlated with seedling biomass: *Peziza depressa*, *Clavulina cirrhata*, *Cenococcum geophilum*, *Inocybe fibrillosibrunnea*, and *Cortinarius amoenus*. The presence of *P. depressa* and *I. fibrillosibrunnea* were significantly related to seedling biomass, while *C. amoenus* was marginally related to seedling biomass (Table 3). The presence of *P. depressa* was correlated with higher mean seedling biomass (Table 3). *Peziza depressa* was most abundant in DR areas (Table S2). Conversely, the presences of *I. fibrillosibrunnea* and *C. amoenus* were correlated to lower mean seedling biomass (Table 3) and these OTUs occurred in the PF and AR but not the DR sites (Table S2).

**Table 3:** ANOVA comparison of the relationships between the presence of five EMF taxa on mean seedling biomass. These five OTUs were highly correlated with NMDS Axis 2, the same ordination axis correlated with seedling biomass. Bolded OTUs indicate significant effects on seedling biomass.

OTU	Species	Sum squares	OTU absent	OTU present	df	*F*	*p*
			Biomass ± S.E.	Biomass ± S.E.			
**1**	*Peziza depressa*	4.92	0.20 ± 0.02	0.41 ± 0.05	1	28.65	0.00
151	*Clavulina cirrhata*	0.41	0.23 ± 0.02	0.29 ± 0.06	1	2.40	0.12
19	*Cenococcum geophilum*	0.10	0.23 ± 0.02	0.09 ± 0.02	1	0.58	0.45
**20**	*Inocybe fibrillosibrunnea*	0.80	0.23 ± 0.02	0.06 ± 0.01	1	4.66	0.03
**55**	*Cortinarius amoenus*	0.65	0.24 ± 0.02	0.07 ± 0.01	1	3.81	0.05

## Discussion

Forest management for high mycorrhizal inoculum potential provides an important aid in forest recovery after harvesting (Perry, Molina & Amaranthus, 1987; Marx, Marrs & Cordell, 2002). We investigated how VR management impacted seedling-EMF interactions after timber harvest and whether it supported EMF dynamics similar to those found in PF stands. In our study we observed a reduction in EMF root colonization and taxonomic richness along with distinct fungal community composition in DR sites compared to PF and AR sites, which shared similar EMF attributes. Overall, EMF composition was significantly correlated with soil moisture and seedling biomass; however, the effects of EMF metrics on seedling biomass were not as important as the management treatment itself. In DR sites, seedlings had greater biomass, and there was greater soil moisture. Together, these findings suggest a stronger abiotic filter, such as soil moisture, on seedling performance than the biotic filter of plant-fungal interactions. Yet, concurrent with reduced EMF metrics in DR sites we also observed reduced recruitment leading us to hypothesize that EMF dynamics may play a role in seedling establishment beyond what we detected by analyzing seedling biomass. However, future experimental studies are necessary to quantify any effects VR may have on seedling recruitment due to EMF attributes. Overall, we observed that AR more than DR treatments maintained EMF diversity in the VR managed forests that we studied.

### EMF composition in relation to other Southern Hemisphere studies

The most abundant EMF taxa observed in our study were ascomyceteous fungi of the order Pezizales. However, the basidiomycetes were more speciose than the ascomycetes. Within the Basidiomycota, the most species-rich order and family were the Agaricales and the Cortinariaceae, respectively. These patterns of taxonomic diversity mirror those in surveys of both root tips (Nouhra et al., 2013) and fungal fruiting bodies in the *Nothofagus* forests of South America (Garnica, Weiß& Oberwinkler, 2003; Truong et al., 2017). BLAST results for the five most abundant OTUs in our EMF dataset matched taxa observed in Chile and Argentina. Some of these taxa were previously thought to only occur in Australasia (e.g., OTU1, top BLAST description *Ruhlandiella* sp. (Truong et al., 2017)). Interestingly, the dominant families (Cortinariaceae, Inocybaceae, and Thelephoraceae) observed in our root tip survey are also abundant mycobionts in Australian forests (Tedersoo et al., 2009; Horton et al., 2017), and the most species-rich families observed at high latitudes in the Northern Hemisphere (Timling et al., 2012). Our sequence dataset supports recently described patterns of EMF diversity in South America (Truong et al., 2017) and draws attention again (Tedersoo et al., 2008) to parallels between EMF taxonomic diversity at high latitudes in both the Northern and Southern Hemispheres.

### VR impacts on EMF

We found that *N. pumilio* seedling root systems were highly colonized by EMF (∼97% across all the studied treatments). These findings are similar to reports that both young and mature *Nothofagus* roots have high colonization levels of >70% (Diehl, Mazzarino & Fontenla, 2008; Fernández et al., 2015) and reinforce findings from the Northern Hemisphere where inoculum levels in logged areas remain high (Perry et al., 1982; Jones, Durall & Cairney, 2003) even when strong differences in fungal composition occur after harvest. Despite overall high colonization levels, mycorrhizal colonization was lowest in DR sites akin to observations of reduced EMF root presence in harvested New Zealand *Nothofagus* forests (Dickie, Richardson & Wiser, 2009). Nonetheless, even in DR sites, colonization levels were ∼95%, indicating that EMF inoculum was not severely limited for seedlings that recruited successfully.

Dispersed retention sites supported lower α diversity than PF and AR sites. Several studies from the Northern Hemisphere report reduced EMF richness with increasing distance from the forest edge or individual green trees for both seedlings that naturally established before logging (advanced regeneration) and experimentally established seedlings (Kranabetter, 1999; Hagerman, Sakakibara & Durall, 2001; Kranabetter & Friesen, 2002; Outerbridge & Trofymow, 2004; Teste, Simard & Durall, 2009). We did not directly measure the impact of seedling proximity to seed trees or the forest edge in the DR sites. Instead our results show that, on average, the DR areas have reduced EMF richness compared to the other treatments. This does not exclude the possibility that there are mycorrhizal hot-spots within DR areas due to proximity to mature tree rooting zones; however, our measurements of EMF attributes were not highly variable from seedling to seedling in the DR, which we would expect if there were great contrasts in EMF dynamics within versus beyond the rooting zone of seed trees. The low density of seed trees in the DR area may therefore account for the lower EMF richness on seedlings.

We observed that more taxa were shared between the PF and AR treatments than between PF and DR, supporting the idea that the AR patches act as refugia for species diversity (Soler et al., 2015) more effectively than the individual seed trees in the DR. Although, EMF composition was similar between PF and AR sites and differed in DR sites, there were only two taxa that showed strong gradients in abundance related to VR treatment (*Timgrovea ferruginea* and *Cortinarius cycneus*). Both of these fungi have been observed in environmental sampling in the Southern Hemisphere, but their individual ecologies are not well known (Danks, Lebel & Vernes, 2010; Johnston et al., 2010; Nouhra et al., 2013).

### EMF effects on seedling performance

We detected species-level correlations between five individual EMF taxa and seedling biomass, corroborating findings from greenhouse and field studies demonstrating that the loss or gain of an EMF taxon can influence seedling performance (Jones, Durall & Cairney, 2003 and references therein). We also observed significant correlations between soil moisture, seedling biomass, and EMF community composition. Yet the causality of these relationships is unclear. Monitoring at the permanent PEBANPA network sites shows that DR areas have overall lower seedling and sapling density, which may release seedlings from competition resulting in overall larger seedlings (Martínez Pastur et al., 2009; Martínez Pastur et al., 2011). These larger seedlings may in turn select for particular fungi. Alternatively, the EMF in DR sites may promote greater seedling growth as has been observed in early-successional northern forests (Kranabetter, 2004). Outside of the intraspecific competition dynamics between seedlings and their EMF, the differences in soil moisture related to VR treatment could be a driving factor in explaining the variation in EMF composition (Boucher & Malajczuk, 1990), as well as the positive correlation between EMF and host plant biomass in sites with higher moisture (Kennedy & Peay, 2007). Yet a fourth explanation of these correlations between seedling size, recruitment density, and EMF may be the strong abiotic gradients across the VR treatments (Martínez Pastur et al., 2007; Martínez Pastur et al., 2011; Martínez Pastur et al., 2014). The high light and moisture levels in DR, compared to AR and PF sites may support relatively high biomass accrual when seedlings do recruit successfully. However, the consistently lower recruitment levels regardless of seed availability and good seedbed conditions (Martínez Pastur et al., 2011; Torres et al., 2015; Table 1) in DR sites may be due in part to reduced EMF metrics, and we therefore suggest that EMF may play a larger role in recruitment than we were able to measure with our biomass metric. Further experimentation is needed to disentangle the hierarchy of VR effects on the outcomes of seedling-EMF interactions (*sensu* Kranabetter, 2004; Nicholson & Jones, 2017).

### VR impacts and conservation of forest biodiversity

Our study of the VR management regime can be used to infer how forests managed for multiple goals impact the diversity of EMF and plant-fungal interactions at the seedling phase in southern *Nothofagus* forests. Unlike other studies from our study region that have found that AR and DR sites increase the species richness of various taxa of insects, birds and plants (Soler et al., 2015; Soler et al., 2016), we found that AR sites maintained EMF richness and DR supported a lower number of taxa. The contradiction with other observations in our study system of VR effects on biodiversity likely has to do with the obligate symbiosis of EMF with *N. pumilio* host plants and is supported by findings from EMF studies in the Northern Hemisphere (Kranabetter, De Montigny & Ross, 2013). Within our study region, Soler et al. (2015) found that AR maintained the forest structure and microenvironment conditions found in PF stands. With variation in the physical environment more similar between PF and AR than AR and DR or PF and DR, it follows that treatment-level α diversity, evenness, and root colonization would vary more significantly between DR and the other treatments. The DR treatment negatively affected forest structure (Soler et al., 2015), resulting in a reduction in EMF host plants. Therefore, the shift in EMF composition between treatments is likely due to the presence and abundance of adult trees and may be further modulated by EMF dispersal capabilities (Peay et al., 2012; Horton, 2017), competitive abilities (Kennedy et al., 2007; Kennedy et al., 2011), and disturbance tolerance of EMF present in DR sites. A study of the impacts of dispersed green tree retention and AR showed that together these treatments maintained sporocarp production of EMF (Luoma et al., 2004), while in our study AR seem most important for the maintenance of EMF community dynamics.

## Conclusions

The VR management applied to *N. pumilio* forests had a clear impact on recruitment metrics like seedling density and biomass and the structure of EMF communities. The DR sites had higher soil moisture and fewer seedlings, but the seedlings had greater biomass along with lower EMF diversity, colonization and different composition than seedlings in AR and PF stands. In contrast, EMF richness, taxonomic diversity, and composition were maintained in AR compared to PF stands and are likely important to seedling recruitment. However, the shifts in EMF attributes across VR treatments were not the most important variables explaining variation in seedling biomass. This suggests that the effects of EMF post-harvest on seedling biomass may be secondary to or muted by other, presumably abiotic, variables, such as seedling access to resources like soil moisture, which has been shown to be important to seedling success in our study area. Our results provide both additional support for the notion that AR sites act as islands of ecological integrity, maintaining biological legacies from PF stands, and highlight the effects of the complex interactions between retention treatments, the post-harvest abiotic environment, and EMF on seedling recruitment dynamics.

##  Supplemental Information

10.7717/peerj.5008/supp-1 Table S1 Operational Taxonomic Units (OTUs) detected on the root systems of seedlings at Los Cerros Ranch Phylum B = Basidiomycota, A = Ascomycota. Guild EMF = ectomycorrhizal fungus, DSE = dark septate endophyte, ERM = ericoid mycorrhizal fungus, SAP = saprotrophic fungus. Reference and Identity (%) is the sequence similarity between the query sequence and the hit sequence from the GenBank database. Coverage is the percentage of the top match sequence that aligns with the query sequence.

10.7717/peerj.5008/supp-2 Table S2 Sequence abundances and distributions of ectomycorrhizal fungi at Los Cerros Ranch Species identities from RDP Naive Bayesian assignment and from the top Blast match.

10.7717/peerj.5008/supp-3 Figure S1 NMS ordination (Axis 1 and 2) of EMF communities associated with each seedling Symbols represent each variable retention timber treatment. Blue circles = Aggregate retention, orange squares = Dispersed retention, and black triangles = Primary forest. Proportion of variance explained by each axis: Axis 1 = 29.2%, Axis 2 = 22.5%, and Axis 3 = 13.2% of the variation in EMF composition. The NMDS ordination had a final stress of 10.86 indicating that the ordination did a fair job of representing fungal compositional patterns and had an acceptable final instability of 0.00048 after 381 iterations. Gradients (axes) were exported and used in Random Forest and mixed model analysis to represent EMF composition.

10.7717/peerj.5008/supp-4 Supplemental Information 1 Ectomycorrhizal community composition associated with *Nothofagus pumilio* seedlings harvested from Variable Retention treatments at Los Cerros Ranch, Tierra del Fuego, Argentina Each data point represents operational taxonomic unit abundance for each seedling.

10.7717/peerj.5008/supp-5 Supplemental Information 2 Mycorrhizal colonization, age, and aboveground biomass (g) of *Nothofagus pumilio* seedlings harvested from Variable Retention treatments at Los Cerros Ranch, Tierra del Fuego, Argentina Each data point provides the raw data assoicated with each seedling harvested in aggregate retention, dispersed retnetnion, or primary forest stands.

## Additional Information and Declarations

### DNA Deposition

The following information was supplied regarding the deposition of DNA sequences: The fungal sequences have been submitted to GenBank under accession numbers MH019772 –MH019959.

### Data Availability

The following information was supplied regarding data availability: Hewitt, Rebecca Eliza; Taylor, D Lee; Hollingsworth, Teresa Nettleton; Martinez Pastur, Guillermo; Anderson, Christopher B. 2018. Ectomycorrhizal community composition associated with *Nothofagus pumilio* seedlings harvested from Variable Retention treatments at Los Cerros Ranch, Tierra del Fuego, Argentina. Bonanza Creek LTER—University of Alaska Fairbanks. BNZ:686, http://www.lter.uaf.edu/data/data-detail/id/686. doi: 10.6073/pasta/df801ec9b7f3493b6eb3bf191124e41f. Hewitt, Rebecca Eliza; Taylor, D Lee; Hollingsworth, Teresa Nettleton; Martinez Pastur, Guillermo; Anderson, Christopher B 2018. Mycorrhizal colonization, age, and aboveground biomass (g) of *Nothofagus pumilio* seedlings harvested from Variable Retention treatments at Los Cerros Ranch, Tierra del Fuego, Argentina. Bonanza Creek LTER—University of Alaska Fairbanks. BNZ:687, http://www.lter.uaf.edu/data/data-detail/id/687. doi:10.6073/pasta/350b701beb4ac2ebd2ef3f29893e6ed5. These files are also available as Supplemental Files.
