# Supplementary figures and images for: Variable retention harvesting influences belowground plant-fungal interactions of Nothofagus pumilio seedlings in forests of southern Patagonia
Source: PeerJ. 2018 Jul 6;6:e5008. doi: 10.7717/peerj.5008 (PMC6037133; doi:10.7717/peerj.5008)

Axis 2

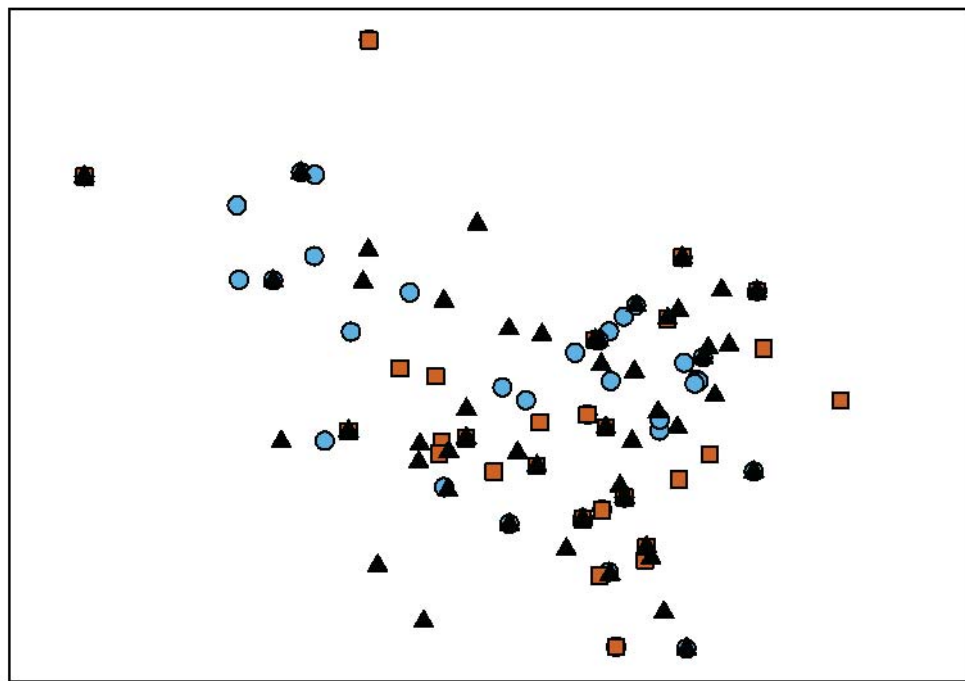

Axis 1

Supplement: Figure S1 — Symbols represent each variable retention timber treatment. Blue circles = Aggregate retention, orange squares = Dispersed retention, and black triangles = Primary forest. Proportion of variance explained by each axis: Axis 1 = 29.2%, Axis 2 = 22.5%, and Axis 3 = 13.2% of the variation in EMF composition. The NMDS ordination had a final stress of 10.86 indicating that the ordination did a fair job of representing fungal compositional patterns and had an acceptable final instability of 0.00048 after 381 iterations. Gradients (axes) were exported and used in Random Forest and mixed model analysis to represent EMF composition. [file peerj-06-5008-s003.pdf]
